# Impact electrochemistry reveals that graphene nanoplatelets catalyse the oxidation of dopamine *via* adsorption†
†Electronic supplementary information (ESI) available. See DOI: 10.1039/c7sc03672h


**DOI:** 10.1039/c7sc03672h

**Published:** 2017-10-30

**Authors:** Lifu Chen, Eden E. L. Tanner, Chuhong Lin, Richard G. Compton

**Affiliations:** a Department of Chemistry, Physical and Theoretical Chemistry Laboratory , University of Oxford , South Parks Road , Oxford OX1 3QZ , UK . Email: richard.compton@chem.ox.ac.uk ; Fax: +44 (0)1865 275410 ; Tel: +44 (0)1865 275957

## Abstract

Graphene nanoplatelets are shown to electrocatalyse the oxidation of dopamine. Single entity measurements (‘nano-impacts’) coupled with microdisc voltammetry and UV-visible spectroscopy reveal that adsorption of dopamine and its oxidised product on the graphene nanoplatelets is the key factor causing the observed catalysis. Genetic implications are drawn both for the study of catalysts in general and for graphene nanoplatelets in particular.

## Abstract 2

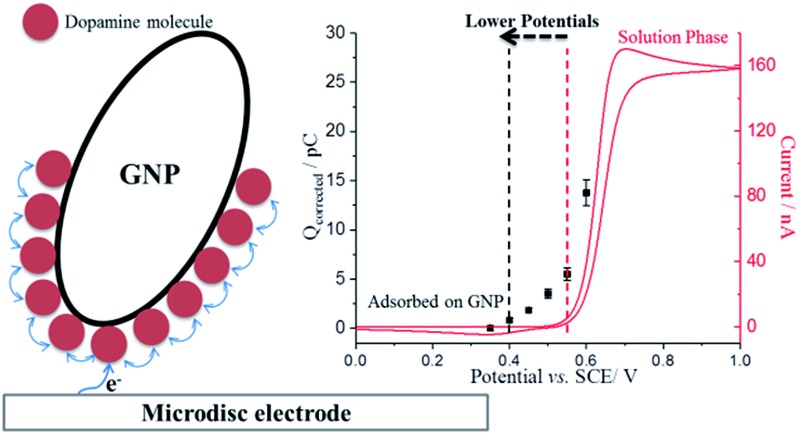
Single entity measurements (‘nano-impacts’) reveal that adsorption of dopamine and its oxidised product on the graphene is the key factor causing the observed catalysis.

## Introduction

Graphene nanoplatelets (GNPs) have been proposed as effective electrocatalysts for various redox processes including some which lie near the heart of energy transformation technology such as solar cells.^1^–^7^ The GNPs are typically applied as layers or ensembles on other electrodes acting as a substrate with the observed electrochemistry showing decreases in the potential required for the oxidation or reduction process of interest.^8^ However, the mechanism for GNP catalysis or electrocatalysis has received relatively little attention hitherto.

In the present paper, we examine the oxidation of dopamine in aqueous acid in the presence and absence of GNPs with the aim of identifying any catalysis and understanding the origin of this effect in physicochemical terms. Dopamine is used as a model redox active adsorbate which plays important roles in biomedical applications^9^ and dye-sensitized solar cells.^10^ To this end we utilise the nano-impacts technique so as to allow the observation of the electrochemistry of dopamine at single GNPs. In this method^11^–^14^ the particles are suspended in solution but from time to time collide with (impact) a microelectrode held at a suitable electrical potential. For the duration of the impact the conducting particle may act as a tiny electrode with the same potential of the impacted microelectrode and hence electrochemistry can be observed exclusively during the duration of the impact if the redox process studied occurs selectively on the particle rather than the electrode.^15^,^16^ Thus by careful choice of the electrode material, the electrochemistry at single particles can be observed. In particular if an electrochemical response is seen during the ‘nano-impact’ and not on the substrate electrode then clearly the process is more favoured – thermodynamically and/or kinetically – on the material of the particle than that of the electrode so providing a very easy, qualitative assessment of the relative catalytic behaviour of the two materials for the process of interest.

Herein we compare the oxidation of dopamine at single entity GNPs with that at glassy carbon (GC). Further, building on previous work,^5^ we explore the role of adsorption in the observed apparent catalysis and investigate possible causes of this using the nano-impacts technique as already applied to GNPs^17^,^18^ for the observation of their properties including their potential of zero charge (PZC) and their diffusion coefficient in aqueous solution.

## Experimental

### Chemicals and reagents

All chemicals were purchased from Sigma-Aldrich at reagent grade unless stated otherwise. The previously characterised^17^ graphene nanoplatelets (15 μm in width, 6–8 nm in thickness) with an average area of 297 ± 152 μm^2^ (estimated from scanning electron microscopy^17^) were acquired from Strem Chemicals (MA, USA). All chemicals were used without further purification. All solutions were made up using ultrapure water of resistivity not less than 18.2 MΩ cm (Millipore, MA, USA) at 298 K. The buffer solution (pH = 0) was freshly prepared from hydrochloric acid (37%) and confirmed by using a Hannah pH231 pH meter (Hannah, Bedfordshire, UK). It has been reported that DA is more stable and water-soluble in the protonated form in acidic environments,^19^ hence all following experiments were conducted at pH 0 (1.0 M HCl). The buffer was degassed thoroughly with pure nitrogen (BOC Gases, UK) for 15 min to prevent degradation of the solution by atmospheric oxygen prior to the addition of DA.

### UV-vis spectroscopy and adsorption measurements

To construct the adsorption isotherm of DA on GNPs, UV-vis studies were performed to accurately measure the uptake of DA by GNPs. A certain amount of GNPs were mixed with a defined unit volume (1.0 mL) of different concentrations of DA solutions. The amount of GNPs used for each DA solution was estimated by calculation such that the GNPs used must adsorb at least 20% of DA in solution, assuming a full monolayer coverage of DA on GNPs. The mixture of GNPs and DA solution was then sonicated (FB15050, Fisher Scientific, 50/60 Hz, 80 W, Germany) for 35 min to allow full adsorption, followed by centrifugation (Eppendorf Centrifuge 5430 R) for 10 min at 14 000 rpm. The original DA solution prior to adsorption and the supernatant after adsorption then centrifugation were both diluted into the calibration region then examined by UV-vis spectroscopy.

UV-vis spectroscopy was conducted using a Shimadzu spectrometer UV-1800 and quartz cells with a 10 mm optical path. In all cases, a baseline correction was conducted prior to any measurement, and the absorbance was recorded from 400–220 nm.

### Preparation of GNP suspensions for nano-impact experiments

3.3 × 10^–13^ M of unmodified stock suspensions were prepared by mixing 2.8 mg of GNPs with 5 mL buffer solution. To generate evenly dispersed suspensions the mixtures were sonicated (FB15050, Fisher Scientific, 50/60 Hz, 80 W, Germany) for 25 min. DA modified GNPs suspensions were also prepared. For modified GNP suspensions *via* pre-exposure, the unmodified GNPs were first sonicated in the buffer solution prior to the addition of DA and left for different time for exposure. For modified GNP suspensions *via* sonication, the unmodified GNPs were sonicated in DA solutions for 25 min to generate evenly dispersed suspensions and promote the adsorption. The above suspensions were used as stock solutions, and diluted for nano-impact experiments. Fresh stock suspensions were prepared daily.

### Electrochemical procedures

All electrochemical experiments were performed at 25 °C inside a Faraday cage with a standard three-electrode system using a μAutolab II potentiostat (Metrohm-Autolab BV, Netherlands) and NOVA 1.10 software. For voltammetric measurements, a carbon microdisc electrode (IJ Cambria Scientific Ltd, UK) or a glassy carbon macroelectrode (3 mm diameter) was used as the working electrode, a saturated calomel electrode (SCE) as the reference electrode (SCE, ALS distributed by BASi, Tokyo, Japan) and a graphite rod as the counter electrode. The carbon microdisc electrode radius was calibrated as 26.5 μm electrochemically by analysing the steady state voltammetry of 1.0 mM hexaamineruthenium(iii) chloride in aqueous solution containing 0.1 M KCl, using a diffusion coefficient for [Ru(NH_3_)_6_]^3+^ of 8.43 × 10^–10^ m^2^ s^–1^ at 298 K.^20^ Prior to each voltammetric experiment, the carbon microdisc electrode and glassy carbon macroelectrode were polished using alumina of decreasing particle size (1.0, 0.3 and 0.05 μm, Buehler, IL, UK) followed by sonication in water and drying with nitrogen. Cyclic voltammetry (CV) was conducted at selected scan rates of between 25 mV s^–1^ to 1000 mV s^–1^ in pH 0 buffer solution.

For nano-impact and chronoamperometry, the same carbon microdisc electrode was used as a working electrode with the same reference and counter electrodes as above. Note that the potentiostat used in this work accurately conserves the charge transferred due to a particle-impact process despite possible alteration in the spike shape.^13^,^21^ 4.5 mL of buffer solution was nitrogen degassed for 5 min to remove dissolved oxygen and 500 μL of unmodified GNPs stock suspension was then added while the nitrogen was kept bubbling for further 5 s to get an even suspension, followed by immediate chronoamperometric scans. For catalytic nano-impact experiments with the present of DA, a known concentration of DA was used with adding of unmodified or modified GNPs. The program “SignalCounter” was used for impact spike identification and individual spike charge determination.^22^

## Results and discussion

This section first reports the adsorption of dopamine (DA) on graphene nanoplatelets (GNPs), characterised by UV-vis spectroscopy. Second, solution phase voltammetry of DA on carbon electrodes was undertaken, followed third, by use of the nano-impact methodology to investigate the oxidation of DA at single GNPs.

### Adsorption of dopamine on GNPs

**Fig. 1:**
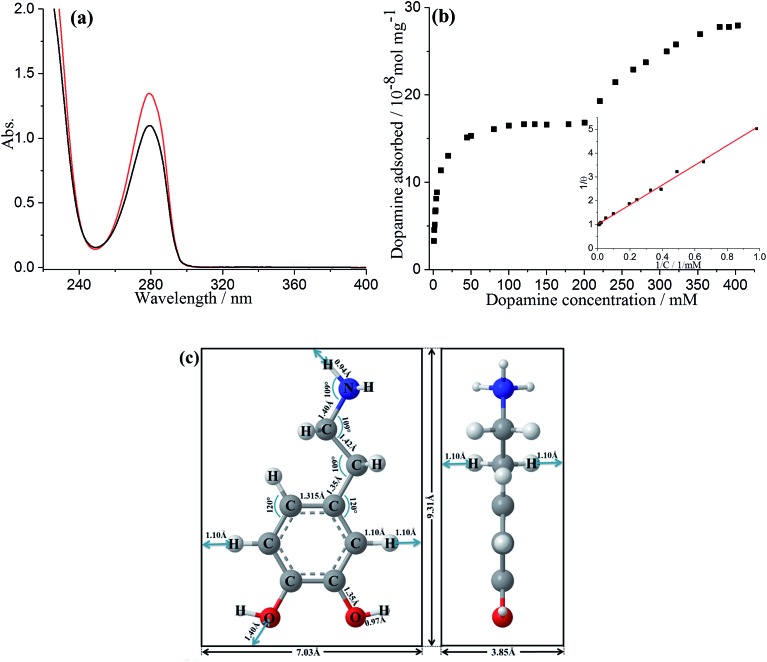
(a) UV-vis absorption of a pH 0 solution containing 5 mM dopamine before GNPs adsorption (red line) and after adsorption (black line); dilution factor = 10. (b) Dopamine adsorption isotherm for GNPs in pH 0 buffer. Inset: Langmuir plot of dopamine on GNPs in pH 0 buffer, where *θ* is the fractional surface coverage and *C* is the adsorbate concentration, Langmuir adsorption model applies when the DA concentration is lower than 200 mM. (c) Rectangular box model of dopamine molecule for flat view (left) and edgewise view (right).

The adsorption of DA on GNPs was first studied by UV-vis spectroscopy. 1 mL of DA solution was mixed with GNPs and sonicated for 35 min to allow full adsorptive uptake, followed by centrifugation. The amount of DA immobilized on the GNPs was quantified by examining the original DA solution before adsorption and the supernatant after adsorption *via* UV-vis spectroscopy. As shown in Fig. 1a, the absorbance peak of dopamine in aqueous solution is at 279 nm, in good agreement with literature.^23^ A linear Beer–Lambert plot was obtained with the extinction coefficient (*ε*) of 0.257 M^–1^ m^–1^ illustrating the relationship between absorbance and DA concentration, as shown in Fig. S1.† It is evident that the reduction in magnitude of absorbance peak results from the adsorption onto GNPs. The adsorption isotherm of dopamine for GNPs in aqueous buffer was then plotted as shown in Fig. 1b. The presence of two distinct plateaux suggest a flat to vertical concentration driven phase transition^24^,^25^ of DA molecules adsorbed on GNPs, corresponding respectively to flat molecular orientation at low concentrations (≤200 mM), and the vertical orientation of DA molecules at higher concentrations (≥353 mM). This was confirmed by comparing the average area occupied by each individual molecule (*S*_R-DA_) on GNPs with the theoretical estimated area of the molecule in each possible orientation. As illustrated in Fig. 1c, the theoretical areas of DA were determined by approximating the DA molecule as a rectangular box with all side lengths estimated by trigonometry for bond lengths, bond angles (obtained from ChemDraw 15.1) and van de Waals radii of terminating atoms (tabulated by Rowland).^26^ The molecule area of flat view (*S*_DA_) and of edgewise view (*S*′_DA_) hence are estimated as 6.5 × 10^–15^ cm^2^ and 3.6 × 10^–15^ cm^2^ respectively, in agreement with literature.^27^ In the dopamine isotherm, the amount of DA adsorbed by a unit amount of GNPs reaches the first plateau at adsorbate concentrations approaching 200 mM with a limiting uptake of 1.7 × 10^–7^ mol mg^–1^, giving the maximum surface coverage (*Γ*_max_) of (2.6 ± 0.8) × 10^–10^ mol cm^–2^ and the average area occupied by each individual molecule (*S*_R-DA_) of (6.4 ± 1.5) × 10^–15^ cm^2^ (see SI for detailed calculations†) consistent with the flat DA molecule area. The second plateau occurs at concentrations approaching 400 mM with a limiting uptake of 2.7 × 10^–7^ mol mg^–1^, *Γ*′_max_ = (4.3 ± 1.3) × 10^–10^ mol cm^–2^ and *S*′_R-DA_ = (3.8 ± 0.9) × 10^–15^ cm^2^, in good agreement with the edgewise (vertical) molecule area. It is also notable that the phase transition of dopamine is less abrupt and the second plateau is reached only at considerably higher adsorbate concentrations as compared with catechol,^24^ in excellent agreement with Hubbard's observation of the adsorption of dopamine on platinum.^28^

The low concentration region (≤200 mM) was then analysed in terms of Langmuir model, which predicts the fractional coverage, *θ*, to vary with adsorbate concentration, *C*,1
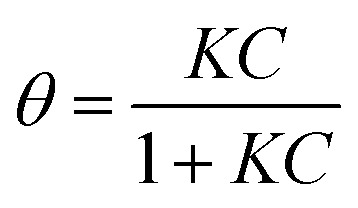
where *θ* = *Γ*/*Γ*_max_ and *Γ* is the coverage corresponding to the adsorbate concentration. As shown in Fig. 1b inset, a straight line with intercept of 1.006 and *R*^2^ = 0.996 is obtained indicating excellent agreement with the Langmuir model, suggesting that the adsorption is reversible. Furthermore, the adsorption constant (*K*) was estimated from the reciprocal of the slope, giving (0.24 ± 0.004) mM^–1^.

### Oxidation of dopamine on micro and macro carbon electrodes

**Scheme 1:**
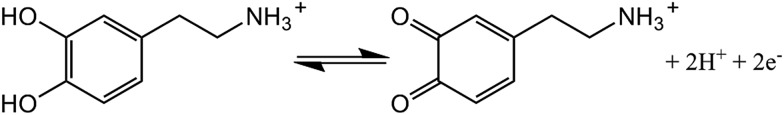
Two-electron, two-proton oxidation of dopamine.

Next, the oxidation of DA in aqueous acid in the presence and absence of GNPs was then examined electrochemically. The cyclic voltammetric responses of bare microdisc carbon electrodes (radius 26.5 μm) were first recorded in pH 0 buffer solution (1.0 M HCl) containing variable DA concentrations (from 0 to 10 mM) at scan rates of 25 mV s^–1^ to 1000 mV s^–1^. The half-wave potential (*E*_1/2_) of the voltammograms is independent of DA concentration with a constant value of *ca.* +0.64 V *vs.* SCE across the range of scan rates (Fig. S2†), suggesting the electrochemical reversibility of the process. Moreover, literature suggests that the voltammetric responses likely correspond to a two-electron, two-proton oxidation of DA^29^ (Scheme 1). Note that the amine group is protonated in the pH 0 environment.^30^

**Fig. 2:**
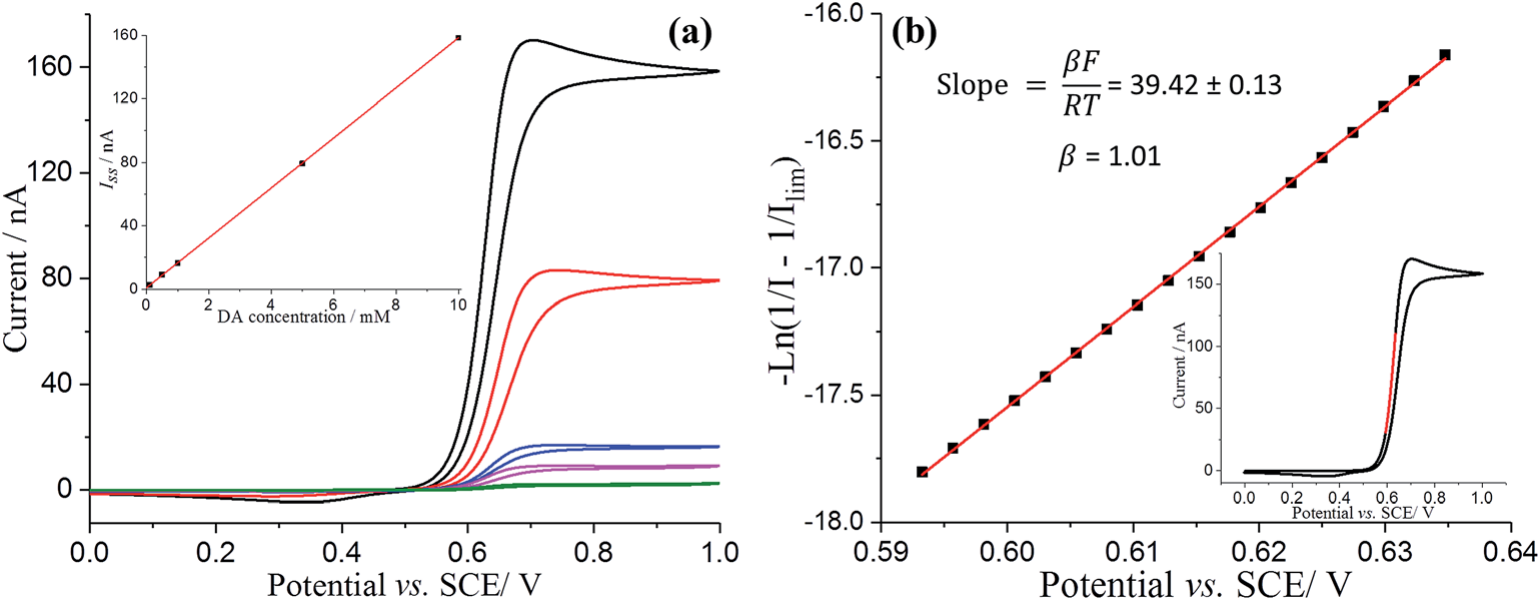
(a) Voltammograms of a bare microdisc carbon electrode in pH 0 buffered solution containing variable concentration of DA (10 mM, black; 5 mM red; 1 mM blue; 0.5 mM magenta; 0.1 mM green) at a scan rate of 25 mV s^–1^. Inset shows the linear correlation of limiting current (*I*_ss_) as a function of DA concentration. (b) Mass-transfer corrected Tafel analysis for voltammogram of a bare microdisc carbon electrode in 10 mM dopamine at scan rate of 25 mV s^–1^. The highlighted red region in inset voltammograms was selected as the Tafel analysis region.

Fig. 2a compares the microdisc voltammetric responses in variable concentrations of DA solutions. The magnitude of the steady-state limiting current (*I*_ss_) of a diffusion-controlled process, assuming that all of DA molecules that diffuse to the electrode surface undergo oxidation, is described by:

2 *I*_ss_ = 4*nFDCr*

where *n* = 2 (the number of electrons transferred), *F* is the Faraday constant, *D* is the diffusion coefficient of DA, *C* is the bulk concentration of DA, and *r* is the radius of the working electrode.

Studies of the recorded *I*_ss_ as a function of DA concentration allowed a good estimation of the DA diffusion coefficient. From the slope of the limiting current (*I*_ss_) against DA concentration (Fig. 2a inset), the diffusion coefficient of DA was determined as (7.70 ± 0.02) × 10^–10^ m^2^ s^–1^, which is in good agreement with previous reports.^31^,^32^

The experimental voltammetry was next analysed using Tafel analysis.^33^,^34^ To enable an accurate apparent oxidative transfer coefficient (*β*) to be measured, a mass transport corrected analysis^35^ for the voltammetry measured on the microdisc was performed by taking the reciprocal of the experimental current with the reciprocal of the limiting current subtracted 
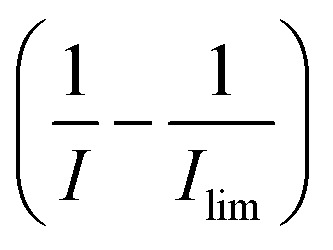
, as shown in Fig. 2b. It is necessary to correct the Tafel analysis to take into account mass transport for the voltammetry on a microelectrode to separate out the electron transfer kinetics from the diffusion. The apparent *β* value determined from the slope is close to unity, suggesting at first sight that the process is a one electron reversible reaction.^36^

**Fig. 3:**
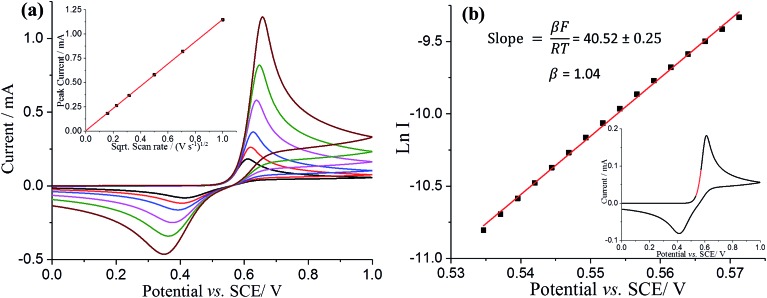
(a) Voltammograms of a bare glassy carbon macroelectrode in pH 0 buffered solution containing 10 mM DA recorded as a function of scan rate (25 mV s^–1^, black line; 50 mV s^–1^, red line; 100 mV s^–1^, red line; 250 mV s^–1^, magenta line; 500 mV s^–1^, green line; 1000 mV s^–1^, brown line). Inset: the plot of peak current as a function of the square root of the scan rate from 25 mV s^–1^ to 1000 mV s^–1^. (b) Tafel analysis for voltammogram of a bare glassy carbon macroelectrode in 10 mM dopamine at a scan rate of 25 mV s^–1^. The highlighted red region in inset voltammograms was selected as the Tafel analysis region.

To reconcile the microelectrode observations, analogous experiments were then conducted with a glassy carbon macroelectrode. The resultant voltammogram showed a single oxidative peak at *ca.* +0.61 V *vs.* SCE and a reductive peak at *ca.* +0.41 V *vs.* SCE (Fig. 3a black line). The peak current was found to be directly proportional to the square root of scan rate, as shown in Fig. 3a inset, suggesting again that the electrochemical process is diffusion controlled. Note that the peak to peak separation is not consistent with a simple one or two electron transfer, reversible or irreversible, suggesting more complex chemistry on the longer timescale of the macroelectrode experiments. In particular the oxidative peak potential shows only a weak scan rate dependence suggestive of a reversible process whilst the reductive peak looks irreversible in character. The existence of follow up chemistry is also indicated by the less positive oxidative peak potential seen on the macroelectrode as compared to the half-wave potential on the microelectrode. This indicates a reversible electron transfer followed by a fast irreversible chemical reaction. Below we interpret the macroelectrode voltammetry in terms of an ECE process so that the back peak is unrelated to the DA/DA^2+^ couple and the lowered potential on the macroelectrode is consistent with a fast chemical reaction following an electrochemically reversible process.

The voltammetry on the macroelectrode is peak shaped, hence we perform a simple Tafel analysis of Ln *I versus E*, but using the part of voltammogram where diffusion plays a near negligible role (Fig. 3b and inset). *β* from Fig. 3b also has value close to unity, suggesting again that the rate determining step comprises a one electron reversible process. This leads us to conclude that overall the oxidation of dopamine is a E_rev_CE process, whereby the first electron transfer is electrochemically reversible and the second electron is fully “driven”. This mechanism will be explored fully in future work.

### Oxidation of dopamine on single GNPs

**Fig. 4:**
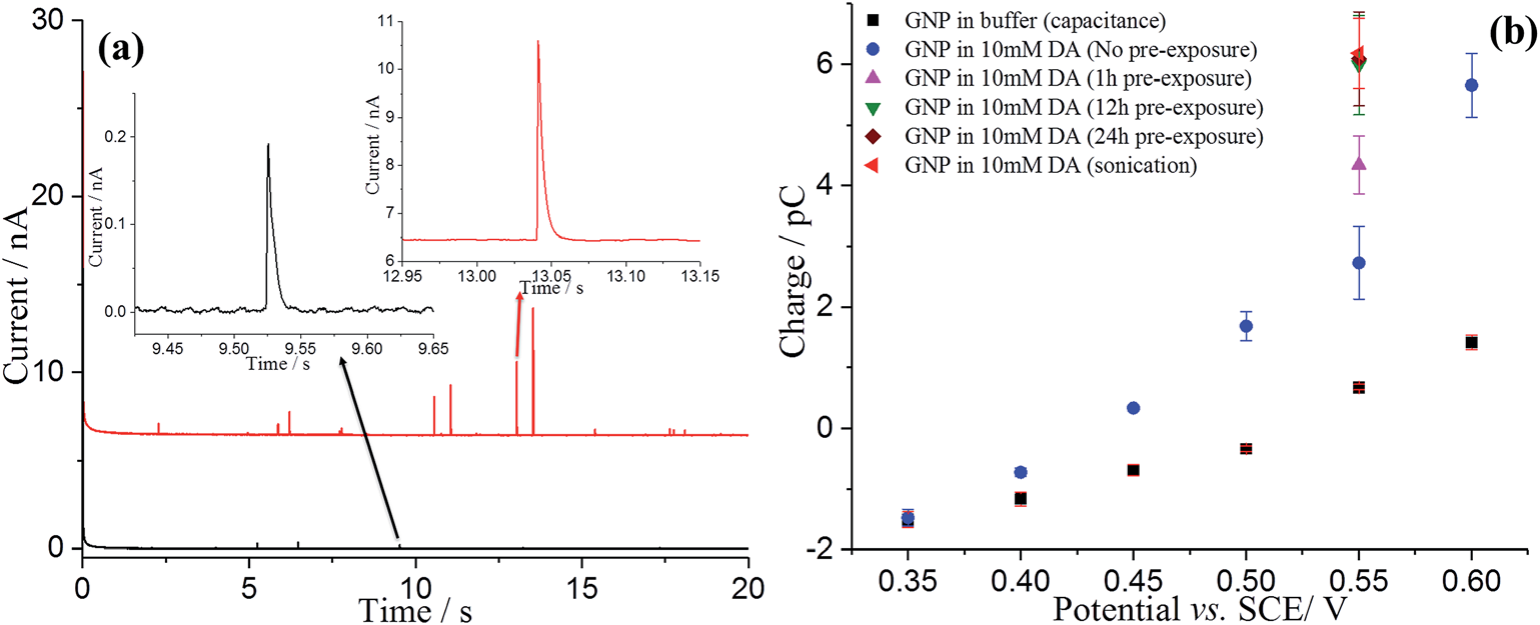
(a) Representative chronoamperometric profiles of nano-impact at +0.55 V *versus* SCE in pH 0 buffer containing: GNPs only (black line); DA-saturated GNPs (modified *via* sonication) and 10 mM DA (red line). (b) Potential variable study of single GNPs: GNPs in buffer only (black), GNPs (no pre-exposure) and 10 mM dopamine (blue). GNPs modified *via* pre-exposure (different exposures time) and sonication in 10 mM DA solutions were also measured at +0.55 V. The error bars are derived from SD/(*n*)^1/2^, where SD is the standard deviation and *n* is the number of the spikes.

The nano-impact method was applied to enable the investigation of the oxidation of dopamine at single GNPs. A carbon microdisc electrode was immersed in a degassed pH 0 buffer solution (1.0 M HCl), and known amounts of dispersed GNPs were added. In the absence of DA, under potentiostatted conditions, clear but small oxidative (capacitative) current spikes at +0.55 V *versus* SCE were detected (Fig. 4a, black). The polarity of the GNPs current spikes^17^ was found to be changed upon alteration of the applied potential to +0.40 V (Fig. S3†), confirming the spikes correspond to capacitative impacts of GNPs. Another control experiment was conducted with no GNPs in the solution; no spikes were detected, further confirming that the occurrence of spikes results from collisions of GNPs with the electrode (Fig. S4†). A potential variation study was then performed and chronoamperograms were recorded at different potentials, from +0.35 to +0.60 V. A plot of average charge of individual capacitative impacts as a function of potential was obtained, as illustrated in Fig. 4b (black squares). The polarity of the spikes changes as a function of potential, consistent with capacitative behaviour. The potential of zero charge (PZC) of GNPs in pH 0 buffer was determined as +0.51 V, as shown in Fig. S5.† The PZC of GNPs in pH 0 buffer is larger than in 0.1 M KCl, 50 mM KH_2_PO_4_, 50 mM K_2_HPO_4_ PBS buffer (–0.14 V, pH = 6.8),^17^ consistent with the pH dependence of the PZC.^37^

**Fig. 5:**
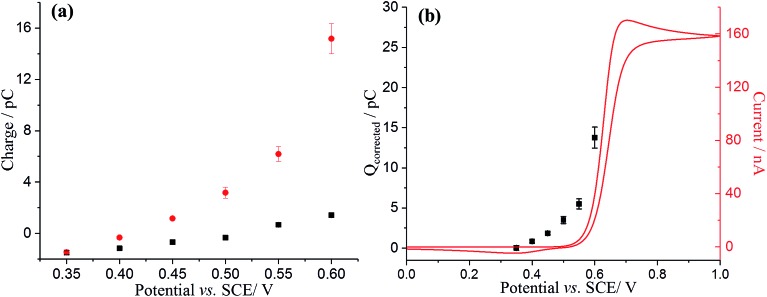
(a) Potential variable study of single GNPs: GNPs only (black), DA-saturated GNPs (modified *via* sonication) and 10 mM dopamine (red). The error bars are derived from SD/(*n*)^1/2^, where SD is the standard deviation and *n* is the number of the spikes. (b) Voltammogram of a microdisc carbon electrode in pH 0 buffered solution containing 10 mM dopamine at a scan rate of 25 mV s^–1^. Overlayed squares are the average corrected charge transferred per individual impact spike (*Q*_correct_ = *Q*_total_ – *Q*_capacitative_) of GNPs at a microdisc electrode.

Analogous nano-impacts experiments of GNPs were conducted in buffer solution containing 10 mM DA. At lower potentials (negative of +0.40 V), the amplitudes of the spikes were similar to those seen without DA, whereas the amplitudes of the spikes increased significantly when the applied potential more positive than +0.45 V (Fig. 4), suggesting that DA is involved in charge transfer and oxidised when a single GNP collides with the electrode at a sufficiently high potential. To further investigate the oxidation reaction of DA catalysed by GNPs, the average charge of individual spikes at a range of potentials was determined and then plotted as a function of potentials (Fig. 4b, blue circles). Comparing this to the capacitative behaviour of single GNPs seen in the absence of DA (black squares), the average charge at each potential is very significantly larger at potentials above +0.45 V, indicating that at sufficiently positive potentials the oxidation of DA accompanied the capacitative charge of single GNPs, contributing to the injection of the measured charge when individual GNPs collide with the electrode. Fig. 5b compares the potential dependence of the impact charge with the voltammetry seen for 10 mM DA on the carbon microdisc electrode in the absence of GNPs. It is apparent that the GNPs likely catalyse the oxidation since appreciable currents flow at potential where DA is not electroactive on glassy carbon. The DA oxidation may likely result from adsorbed DA molecules on GNPs rather than free molecules in aqueous phase, which was further investigated by experiments conducted in 10 mM DA solution with GNPs modified with 10 mM DA solution by pre-exposure or sonication (Fig. 4b). The impact charge of GNPs at +0.55 V increased with an increasing exposure time to the DA solution and saturated for 12 h or longer pre-exposure and sonicated in the DA solution, confirming the oxidation of adsorbed DA on GNPs. Potential variation studies were then conducted with DA-saturated GNPs in 10 mM DA solution (Fig. 5a, red dots). The spike shapes were analysed statistically (Table S1†), showing the majority has a sharp on-and-off shape corresponding to single GNPs colliding with and leaving the electrode.

To further confirm the catalysis of DA oxidation by GNPs, the corrected charge transferred per individual impact spike (corresponding to the faradaic charge) was calculated and compared with the voltammetric response of DA in solution phase at the same microdisc electrode. The switch on potential of DA oxidation decreases from *ca.* +0.52 V *vs.* SCE in the solution phase (Fig. 5b, red line) to *ca.* +0.40 V in the adsorbed DA on GNPs (Fig. 5b, black squares). The origin of this potential shift of *ca.* 120 mV likely reflects the fact that the product (dopamine-*o*-quinone) is more strongly adsorbed on GNPs than on the microdisc electrode, resulting in a more favoured oxidation of DA, since if both the GNP and microdisc signals were solution phase processes the greater mass transport rate to/from the GNPs would shift the potential to more positive potentials which is the opposite of what is observed.

**Fig. 6:**
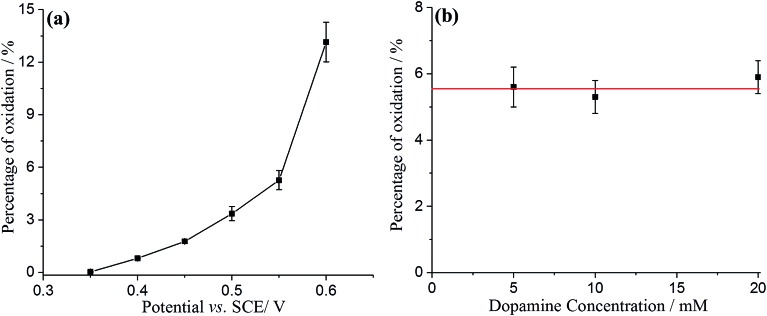
The percentage of adsorbed dopamine on GNPs oxidised during the impact between GNPs and electrode as a function of (a) applied potential; (b) dopamine concentration.

**Scheme 2:**
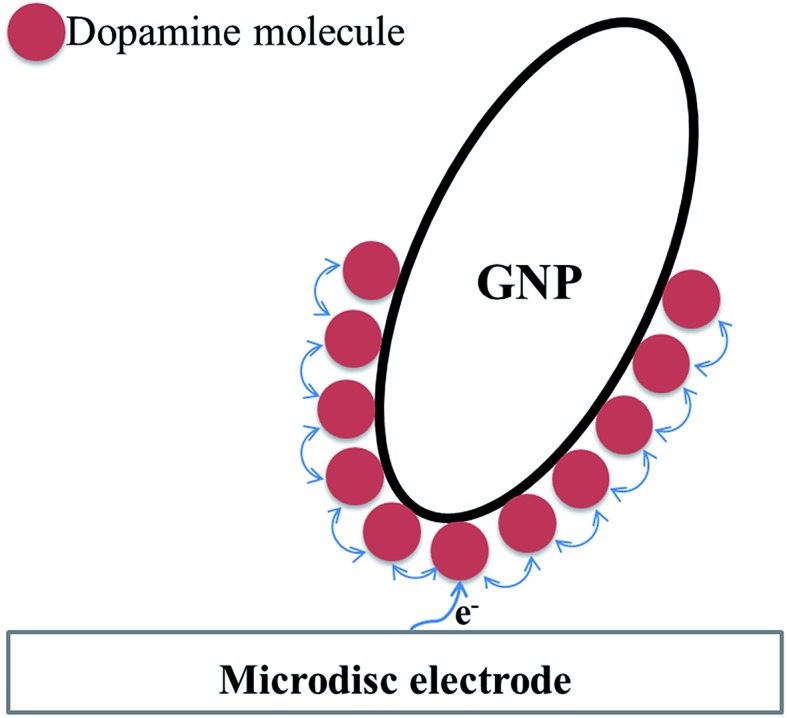
Model of charge diffusion over the surface of GNPs.

Comparing the surface coverage of adsorbed DA on GNPs from UV-vis with the surface coverage of oxidised product on GNPs after impact (see Tables S2 and S3†), a partial oxidation of adsorbed DA on GNPs occurs at low overpotentials. As shown in Fig. 6a, the percentage of oxidation was determined and shown to increase progressively with the potential. Analogous nano-impacts experiments were conducted in different DA concentrations at +0.55 V to compare the percentage of oxidation of adsorbed DA on GNPs with variable surface coverage corresponding to DA solution concentration. Fig. 6b shows that even at variable concentrations of dopamine (and therefore different surface coverages), a consistent 5.6 ± 0.5% of the dopamine undergoes oxidation. The partial oxidation of DA suggests that the oxidation blocks further movement of electrons through the platelet, indicating that the electrons possibly transfer around the surface of instead of through the GNPs, as shown in Scheme 2 and that the adsorbed oxidised product prevents further charge transfer either across the GNP surface or from the electrode.

**Fig. 7:**
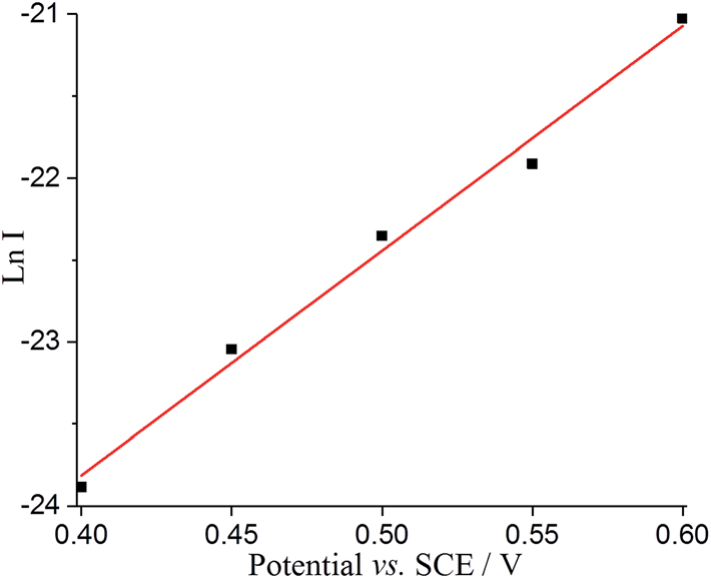
Tafel analysis from nanoimpacts comparing the current of the nanoimpact at different potentials.

Tafel analysis was then performed with the nano-impact data to derive the oxidative transfer coefficient (*β*) for the adsorbed DA oxidation. The impact current (*I*) can be estimated by *I* = *Q*/*t*, where *Q* is the average faradaic charge and *t* is the average impact duration, as shown in Table S4.† As shown in Fig. 7, Ln *I* was plotted as a function of potential, resulting in a linear slope with a value of 13.70 ± 0.78 V^–1^. The transfer coefficient, *β*, can hence be estimated as 0.35 ± 0.02, suggesting the oxidation of adsorbed species on GNP is not reversible and undergoes slow electron transfer kinetics. This further confirms the fact that the spikes in current occur as a result of adsorbed dopamine at a lower potential rather than the solution phase dopamine (as is seen on the micro and macro disc electrodes), allowing the GNPs to catalyse the dopamine oxidation.

## Conclusions

Graphene nanoplatelets were modified with dopamine to allow the investigation of dopamine oxidation at a single GNP by nano-impacts. Compared with solution phase voltammetry on carbon micro and macro disc electrodes whereby the electron transfer is reversible, the irreversible oxidation of dopamine onset at *ca.* +0.40 V for adsorbed dopamine (*vs. ca.* +0.52 V *vs.* SCE for solution phase dopamine). This catalysis is attributed to the increased affinity for the oxidative product, dopamine-*o*-quinone, for the nanoplatelet over the larger electrodes. A model was proposed to explain the incomplete oxidation of the adsorbed dopamine, whereby electron transfer is restricted to surface molecules.

## Conflicts of interest

There are no conflicts to declare.

## Supplementary Material

Supplementary information
