# Supplementary material for: Impact electrochemistry reveals that graphene nanoplatelets catalyse the oxidation of dopamine via adsorption
Source: Chem Sci. 2017 Oct 30;9(1):152–9. doi: 10.1039/c7sc03672h (PMC5869317; doi:10.1039/c7sc03672h)
Supplement: Supplementary file 1 [file SC-009-C7SC03672H-s001.pdf]

Electronic Supplementary Material (ESI) for Chemical Science.
This journal is © The Royal Society of Chemistry 2017

Supporting Information

Impact Electrochemistry Reveals that Graphene Nanoplatelets Catalyse the Oxidation of Dopamine via Adsorption

Lifu Chen, Eden E. L. Tanner, Chuhong Lin and Richard G. Compton*

Department of Chemistry, Physical and Theoretical Chemistry Laboratory, University of Oxford, South Parks Road, Oxford OX1 3QZ, United Kingdom

*Corresponding Author:

Emails: richard.compton@chem.ox.ac.uk

Phone: +44(0) 1865 275957

Fax: +44 (0) 1865 275410

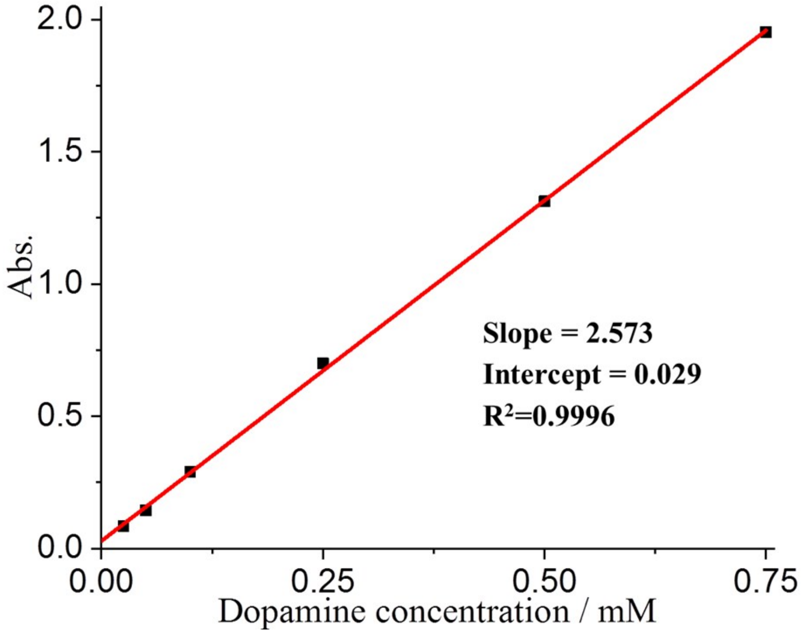

Figure S1. Beer–Lambert plot of dopamine in buffer (pH=0)

The linear relationship between absorbance and absorber concentration can be examined by the Beer-Lambert law.

$$A = \varepsilon\, l\, c$$

Where A is the measured absorbance, ε is the extinction coefficient, l is the path length, and c is the absorber concentration.

$$Slope = \frac{A}{c} = \varepsilon\, l = 2.574\ mM^{-1}$$

$$\varepsilon = \frac{2.574\ mM^{-1}}{l} = \frac{2.574\ mM^{-1}}{10\ mm} = 0.257\ M^{-1}m^{-1}$$

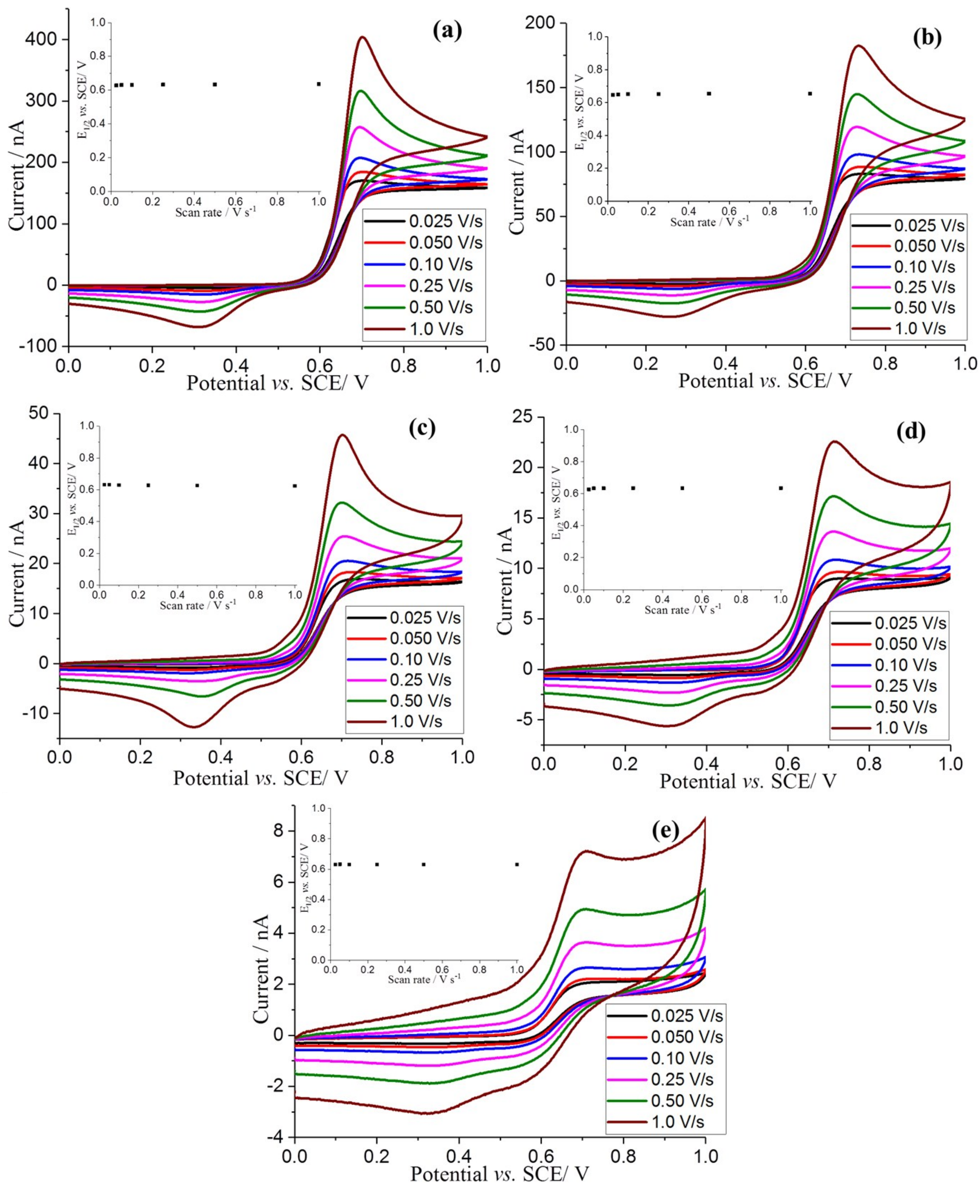

Figure S2 Cyclic voltammograms of a bare microdisc carbon electrode in pH 0 buffered solution containing variable concentrations of dopamine recorded as a function of scan rate from 25 mV s^{-1} to 1000 mV s^{-1}. Inset: the plot of half-wave potential as a function of the scan rate. (a) 10 mM DA, (b) 5 mM DA, (c) 1 mM DA, (d) 0.5 mM DA and (e) 0.1 mM DA.

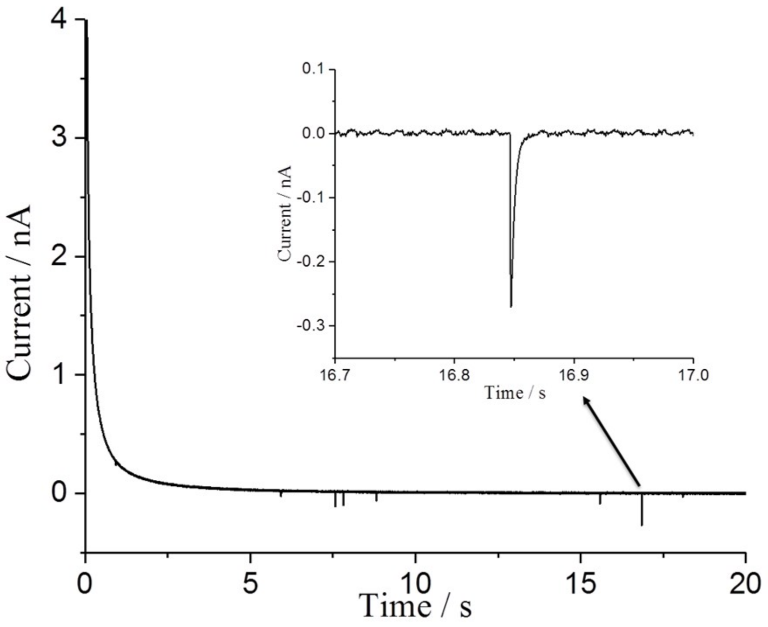

Figure S3. Representative chronoamperometric profiles of nano-impacts at +0.40 V versus SCE in pH 0 buffer containing GNPs only

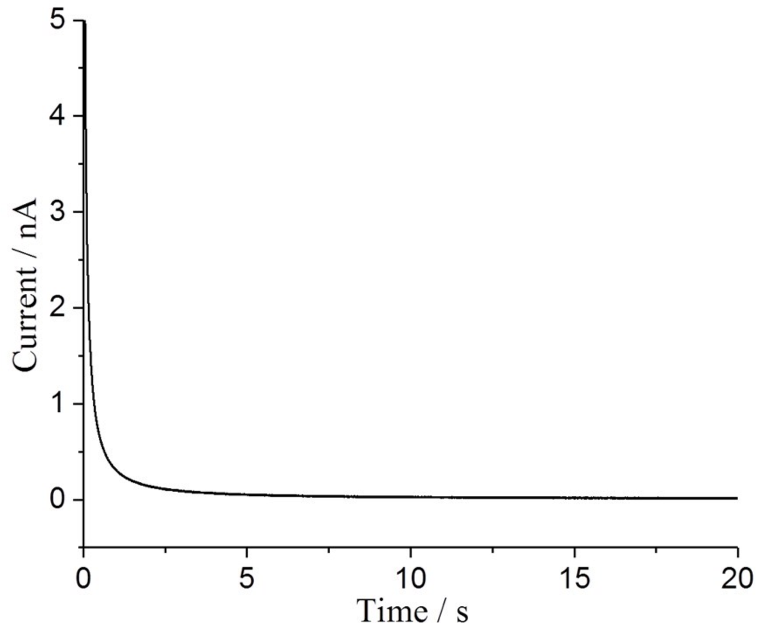

Figure S4. Representative chronoamperometric profiles of nano-impacts at +0.55 V versus SCE in pH 0 buffer without addition of GNPs suspensions

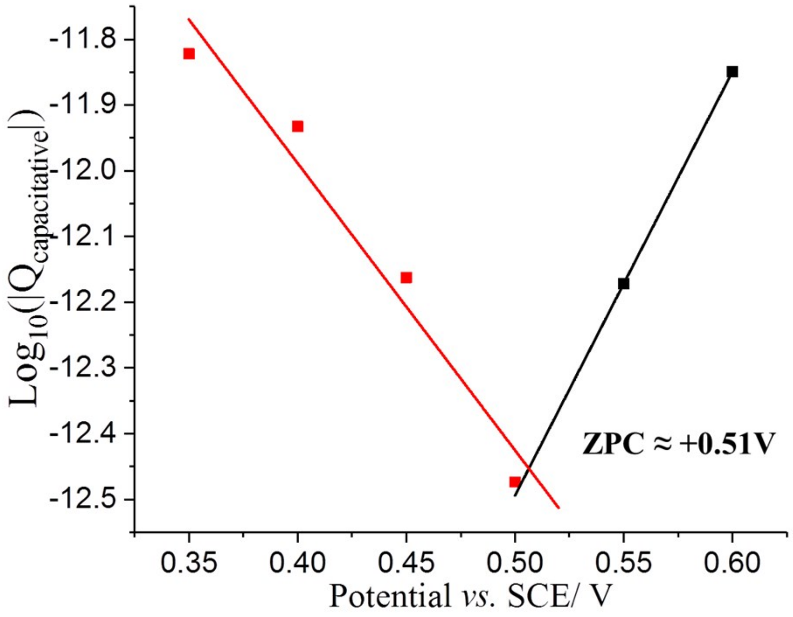

Figure S5. Capacitative impacts with absolute charge in logarithmic ($\log_{10}$) scale plotted against applied potential (vs. SCE). Red squares: values with negative charge; black squares: values with positive charge. The intersection potential of the two lines is used to estimate the PZC value of GNPs at pH 0 environment, where PZC is determined to be +0.51 V (vs. SCE) in pH 0 condition.

Potentials	+0.35V	+0.40V	+0.45V	+0.50V	+0.55V	+0.60V
NOT Sharp	7	6	6	4	3	1
Total	248	146	170	213	193	112
% of Sharp	97%	96%	97%	98%	99%	99%

Table S1. Statistics of spike shape of DA-saturated GNPs (modified via sonication) in 10mM of DA at different potentials

Potential (V)	Γ from UV-Vis of 10mM DA-GNPs (mol / mg of GNP)	Γ from Impact at (mol / mg of GNP)	Percentage of oxidation (%)
+0.35	11.4×10^{-8}	$(3.30 \pm 1.60) \times 10^{-11}$	0.03 ± 0.01
+0.40	11.4×10^{-8}	$(9.17 \pm 0.27) \times 10^{-10}$	0.80 ± 0.02
+0.45	11.4×10^{-8}	$(2.02 \pm 0.11) \times 10^{-9}$	1.8 ± 0.1
+0.50	11.4×10^{-8}	$(3.82 \pm 0.46) \times 10^{-9}$	3.4 ± 0.4
+0.55	11.4×10^{-8}	$(5.99 \pm 0.56) \times 10^{-9}$	5.3 ± 0.5
+0.60	11.4×10^{-8}	$(1.50 \pm 0.12) \times 10^{-8}$	13.1 ± 1.0

Table S2. Summary percentage of adsorbed dopamine during the impact as a function of potential

DA Concentration (mM)	Γ from UV-Vis (mol / mg of GNP)	Γ from Impact at E= 0.55V (mol / mg of GNP)	Percentage of oxidation (%)
5	8.84×10^{-8}	$(4.96 \pm 0.56) \times 10^{-9}$	5.6 ± 0.6
10	11.4×10^{-8}	$(5.99 \pm 0.56) \times 10^{-9}$	5.3 ± 0.5
20	13.0×10^{-8}	$(7.73 \pm 0.65) \times 10^{-9}$	5.9 ± 0.5

Table S3. Summary percentage of adsorbed dopamine during the impact at +0.55 V.

Potentials	+0.35V	+0.40V	+0.45V	+0.50V	+0.55V	+0.60V
Impact time (ms)	25.1	23.2	25.7	23.9	24.8	24.3
Error of mean	1.5	1.9	1.9	1.9	1.3	1.6

Table S4. Summary of average impact times spike shape at different potentials

Adsorption isotherm calculation:

We present an example based on the addition of GNPs to a 5 mM solution of dopamine.

A_1 (absorbance of original 5 mM dopamine solution before GNPs adsorption) = 1.3123

A_2 (absorbance of supernatant after GNPs adsorption) = 1.0655

D (Dilution factor) = 10

The actual concentration of original dopamine solution before GNPs adsorption, c_1, is

$$c_1 = \frac{A_1}{\varepsilon\, l} \times D = \frac{1.3123}{0.257\ M^{-1} m^{-1} \times 10\ mm} \times 10 = 5.10\ mM$$

Similarly, the concentration of supernatant after GNPs adsorption, $c_2 = 4.14\ mM$

10.9 mg of GNPs was used to mix with 1 ml of 5 mM dopamine solution during experiment, hence the amount of DA (n) adsorbed onto 1 mg of GNPs can be determined as

$$n = \frac{(5.10\ mM - 4.14\ mM) \times 1\ ml}{10.9\ mg\ of\ GNPs} = 8.81 \times 10^{-8}\ mol\ of\ DA\ /\ mg\ of\ GNPs$$

Similar calculations are conducted at different dopamine concentrations and consequently the dopamine adsorption isotherm for GNPs in pH 0 buffer is constructed as shown in Figure 1b.

Concentration driven phase transition calculation:

For single GNP particle, average surface area of GNP is 297 ± 152 μm^2 and the thickness is 7.1 ± 2 nm (estimated from scanning electron microscopy[1]). Hence the volume (V) of a single GNP can be estimated as (2.11 ± 1.08) × 10^{-18} m^3. The bulk density of the GNPs is 1 × 10^5 g m^{-3} reported by the supplier.[2] For loose powders, the density of a single GNP is assumed to be the same as graphite, 2.26 × 10^6 g m^{-3}. [3] Therefore, the mass for single GNP can be estimated as (4.77 ± 2.44) × 10^{-9} mg.

The amount of DA (n) adsorbed onto 1 mg of GNPs reaches the first plateau at n_{max} = 1.7 × 10^{-7} mol mg^{-1}. For 1 mg of GNP, the total surface area is:

$$\frac{1}{(4.77 \pm 2.44) \times 10^{-9}\,mg} \times (2.97 \pm 1.52) \times 10^{-6}\,cm^2 = (6.23 \pm 3.19) \times 10^2\,cm^2\,mg^{-1}$$

Therefore, the maximum surface coverage (Γ_{max}) at the first plateau can be determined as:

$$\Gamma_{max} = \frac{1.7 \times 10^{-7}\,mol\,mg^{-1}}{(6.23 \pm 3.19) \times 10^2\,cm^2\,mg^{-1}} = (2.6 \pm 0.8) \times 10^{-10}\,mol\,cm^{-2}$$

The average area occupied by each individual molecule ($S_{\text{R-DA}}$) can be then determined as:

$$S_{R-DA} = \frac{1}{N_A \Gamma_{max}} = \frac{1}{6.022 \times 10^{23}\,mol^{-1} \times (2.6 \pm 0.8) \times 10^{-10}\,mol\,cm^{-2}} = (\ldots \; cm^2$$

A similar calculation is conducted to obtain $S'_{\text{R-DA}}$ at the second plateau.
